# The Pituitary Transcriptional Response Related to Feed Conversion in Pigs

**DOI:** 10.3390/genes10090712

**Published:** 2019-09-14

**Authors:** Katarzyna Piórkowska, Kacper Żukowski, Mirosław Tyra, Magdalena Szyndler-Nędza, Karolina Szulc, Ewa Skrzypczak, Katarzyna Ropka-Molik

**Affiliations:** 1 Department of Animal Molecular Biology, National Research Institute of Animal Production, 31-047 Cracow, Poland; katarzyna.ropka@izoo.krakow.pl; 2 Department of Cattle Breeding, National Research Institute of Animal Production, 31-047 Cracow, Poland; kacper.zukowski@izoo.krakow.pl; 3 Department of Pig Breeding, National Research Institute of Animal Production, 31-047 Cracow, Poland; miroslaw.tyra@izoo.krakow.pl (M.T.); magdalena.szyndler@izoo.krakow.pl (M.S.-N.); 4 Department of Animal Breeding and Product Quality Assessment, Poznań University of Life Sciences, 60-637 Poznań, Poland; karolasz@up.poznan.pl (K.S.); ewa.skrzypczak@up.poznan.pl (E.S.)

**Keywords:** feed conversion, pig, pituitary, prolactin, Wnt signalling

## Abstract

Over the decades, pig breeding objectives have focused on improving the meat content in the carcass without taking into consideration the more effective fattening indicators that affect feed conversion. At present, pig growth traits associated particularly with animal feeding have become crucial due to their economic significance. This is especially evident in countries where pigs are maintained on large farms. The present study indicates that pituitary differentially expressed genes (DEGs) are activated in response to variable feed conversion (FC) in pigs. The experiment included two native Polish breeds: Puławska and Złotnicka White (ZW). The whole pituitary transcriptome was sequenced using next-generation sequencing (NGS) technology. The RNA-seq method identified over 500 and 300 DEGs in the pituitaries of the ZW and the Puławska pig populations, respectively, that were associated with hormonal regulation, notch signaling, and Wnt pathways. Lower FC in the ZW pigs favoured increased fat content in the body and significantly higher prolactin expression. The obtained results indicate that low FC values in pigs are related to slower growth or increased fat content, which suggests various pituitary responses. Therefore, the identified candidate genes were not directly associated with feed conversion values but with other factors. However, the present study delivers new insights into pituitary regulation in pigs.

## 1. Introduction

Over the years, pig breeding objectives have focused on improving the content of meat in the carcass, and significant progress in this area has been achieved [1,2]. In Poland, no actions have yet been undertaken to develop the effectiveness of fattening indicators that impact feed conversion (FC). Today, the largest share of pig maintenance costs, 80%, is for feeding, including forage expenses. A few years ago, pig feeding costs were difficult to estimate due to the ‘homemade’ fodders used in small farms. At present, farm structures have significantly transformed, with the fattening bases of purchased components forming a low share of self-produced cereals. Therefore, feed conversion has become an essential factor in countries where large pig farms are prevalent. Moreover, feed conversion is an economic factor included in breeding programs and is significant in the whole utility model [3].

The pituitary is an overarching endocrine gland controlling various bodily functions and playing a role in balancing hormone levels. It is located in a small bony cavity of the skull below the hypothalamus. The pituitary manages energy balance, the body’s water level and temperature, heartbeat, urination, sleep, hunger, and thirst [4] and is composed of two essential lobes—anterior and posterior. The anterior pituitary has five types of endocrine cells, which secrete thyroid-stimulating hormone (TSH), adrenocorticotropic hormone (ACTH), follicle-stimulating hormone (FSH), luteinizing hormone (LH), growth hormone (GH), and prolactin (PRL). In turn, the posterior lobe releases oxytocin and antidiuretic hormones on demand [5]. The pituitary is believed to be the most important endocrine organ promoting animal postnatal growth [6]. This action is associated with the GH level induced by the growth hormone-releasing hormone (GHRH) signal or the paracrine LH stimulation that modulates GH expression, hormone synthesis, and secretion [7]. Nutritional signals, such as insulin, leptin, insulin-like growth factor I (IGF-I), and specific metabolites, like glucose and free fatty acids (FFA), can modulate GH and LH secretions [8].

In turn, new technology used in molecular biology for next-generation sequencing (NGS) enables a more comprehensive analysis of cell or tissue states. Moreover, due to the NGS, the differentially expressed genes (DEGs) involved in crucial molecular processes can be recognized. The pituitary genes’ expression was examined in various contexts, such as carcinogenesis [9,10], the identification of pituitary stem cells [11], the interpretation of behaviour [12], and the explanation of metabolic disorder bases [13]. Because the pituitary is the predominant gland determining body growth, its transcriptome profiling also served to identify the candidate genes for postnatal development in miniature pigs [14].

The present study aimed to indicate the pituitary candidate genes for feed conversion because it is one of the most important economic factors determining the cost of pig breeding. This research included two native Polish pig breeds, Puławska and Złotnicka White, that are in the national conservation program and were not selected. 

## 2. Materials and Methods 

### 2.1. Ethics Approval and Consent to Participate

The research was performed on biological material derived from pigs maintained and slaughtered in the Pig Test Station (National Research Institute of Animal Production). In the Pig Testing Station, pigs are slaughtered, dissected and, after carcass evaluation, meat is intended for consumption. Therefore, our research does not require the approval of the Animal Experimentation Committee.

### 2.2. Animals

The study included Złotnicka White (ZW, *n* = 16) and Puławska (*n* = 16) female pigs. For each breed, the gilts differed in their feed conversion (FC) and showed high and low feed conversion values. The pigs came from different farms and were unrelated. The tests were performed in the Pig Test Station (PTS) of the National Research Institute of Animal Production in Chorzelów where all animals were maintained under the same housing and feeding conditions. The PTS test began when the pigs reached 30 kg and finished when the pigs reached 100 ± 2.5 kg of body weight and had an age of 184 ± 26 days. During the experiment, the growth traits were recorded according to the PTS procedure. After the trial, the pigs were slaughtered and dissected 24 h later. The pig carcass traits were measured according to Tyra and Żak [15]. Up to 20 min after slaughter, whole pituitaries were collected and stabilized by an RNA-later solution (Ambion, Cambridge, UK), and then they were frozen at −20 °C after 24 h chilling at 4 °C. The differences in body composition between the analysed pig groups with variable FCs were estimated by an ANOVA test (SAS Enterprise v. 7.1 with default settings; SAS Institute, Cary, NC, USA).

### 2.3. cDNA Library Construction 

The whole pituitaries were homogenized with a bead method using Bullet Blender24 (Next, Advance, Troy, MI, USA). The RNA was isolated by a PureLink™ RNA Mini Kit (Ambion, Cambridge, UK) according to the manufacturer’s recommendations. The concentration and quality of the RNA were estimated using TapeStation2200 (Agilent, Palo Alto, CA, USA). The RIN parameter values for all the RNA samples were over 7. The TruSeq RNA Sample Preparation Kit v2 (Illumina, San Diego, CA, USA) was used for cDNA library preparation, with 300 ng of RNA as the input amount according to the supplied protocol. The reverse transcription during the procedure was non-strand-specific. Flowcell clustering was performed using the TruSeq SR Cluster Kit v3-cBot HS (Illumina, San Diego, CA, USA). The sequencing was conducted on a HiScanSQ System (Illumina, San Diego, CA, USA) in single 85 bp cycles (four technical replicates) using TruSeq Kit v3-HS chemistry (Illumina, San Diego, CA, USA), as described by Piórkowska et al. [16].

### 2.4. Raw Read Processing

The raw reads were processed using the FastQC and Flexbar with default parameters. The cleaned reads with a quality higher than 20 (score Q) and more than 36 base pairs were aligned to the Sus_scrofa 11.1 genome assembly (GCA_000003025.6) with reference annotation containing the 22,452 genes listed in the Ensembl database using Bowtie 2 v. 2.3.5.1 and represented by the Fragments Per Kilobase of transcript per Million mapped reads (FPKM) value. The alignment was performed using RNA-Seq by Expectation Maximization (RSEM, v. 1.3.0) supported by the Spliced Transcripts Alignment to a Reference (STAR) aligner, and the statistics were generated by RNA-SeQC v1.1.8; all used default parameters. The sequence data (GSE122343—Puławska, GSE132522—Złotnicka White) has been submitted to the Gene Expression Omnibus (GEO).

### 2.5. Analysis of Differentially Expressed Genes (DEGs)

The analysis of differentially expressed genes (DEGs) was performed using DESeq2 [17] at a false discovery rate (FDR) ≤0.05 (default setting). For each breed, the pituitary DEGs (fold-change ≥1.3) in response to the variable FC were estimated. The STRING functional protein association networks v. 11.0 and PANTHER databases were used for the functional analysis based on the Sus scrofa reference applying FDR ≤ 0.05 correction and default parameters. The gene co-expression within the DEGs was estimated using Cytoscape v. 3.7.1 using a CytoHubba plugin analysis, with the top 5 nodes ranked by a Maximal Clique Centrality (MCC) test and the STRING gene network (default settings).

### 2.6. Validation of RNA-Seq Results and Estimation of Transcript Abundance

The qPCR validation included seven genes and *RPS29* and *OAZ1* as housekeeping controls [18] as well as all the pigs used in the RNA-seq analysis. The genes for validation were chosen based on their relationship to hormonal regulation. Table S1 shows the primers, probes, and assays used in the qPCR method. The similarity between the qPCR and RNA-seq results were calculated using Pearson’s correlation (SAS Enterprise). The gene expression levels were calculated using the delta-delta CT method, as described by Pfaffl [19].

The significant differences between the gene expression levels were estimated using an ANOVA test (Duncan’s post hoc test; SAS Enterprise v. 7.1 with default settings; SAS Institute, Cary, NC, USA).

## 3. Results

### 3.1. Animal Traits 

Both of the investigated pig populations belong to native breeds that are in the national conservation program. In this experiment, pigs were selected based on their feed conversion values, and they were qualified into two feed conversion groups (high feed conversion (HFC) and low feed conversion (LFC)). The fattening measurements showed that, within the Puławska breed, HFC animals took more feed per day compared to the LFC gilts. In ZW pigs, this effect was not observed. In turn, carcass measurements indicated that HFC pigs achieved 100 kg of body mass later, and their ham mass was lower compared to that of the LFC pigs. Within the Złotnicka White group, a higher FC favoured increased fat content. In Puławska pigs, this effect was not observed because the slower-growing pigs were lean (Table 1). 

### 3.2. Analysis of Differentially Expressed Genes 

**Figure 1:**
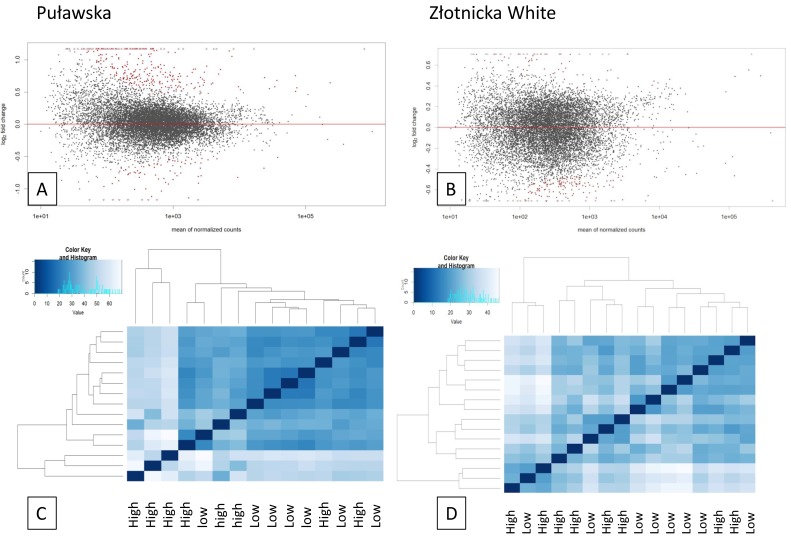
The plots showing the differentially expressed genes (DEGs) in the pig pituitaries identified in the present analysis. The MA (an application of a Bland-Altman plot, M (log ratio) and A (mean average) scales) plots showing several significant (red points) and not significant (black points) results (**A**) for Puławska and (**B**) Złotnicka White pigs. The heatmap Principal Component Analysis (PCA) shows the sample clustering based on the processed reads for (**C**) Puławska and (**D**) Złotnicka breeds characterized by high (HFC) and low feed conversion (LFC).

The statistics are shown in the Supplementary File in Figure S1. After processing, an average of 90% and 80% of the reads (Sus scrofa 11.1) were mapped to the pig reference genome, including 77% and 64% to exonic regions, and 8% and 9% to intronic regions for Puławska and ZW pigs, respectively. The Deseq2 analysis showed that the pituitary response to variable feed conversion was associated with DEGs of 293 and over 500 (a fold-change of ≥1.3 at an adjusted *p*-value ≤ 0.05) (Figure 1) in Puławska and ZW pigs, respectively. DESeq2 results are available at https://bit.ly/2Wtbqlb.

**Figure 2:**
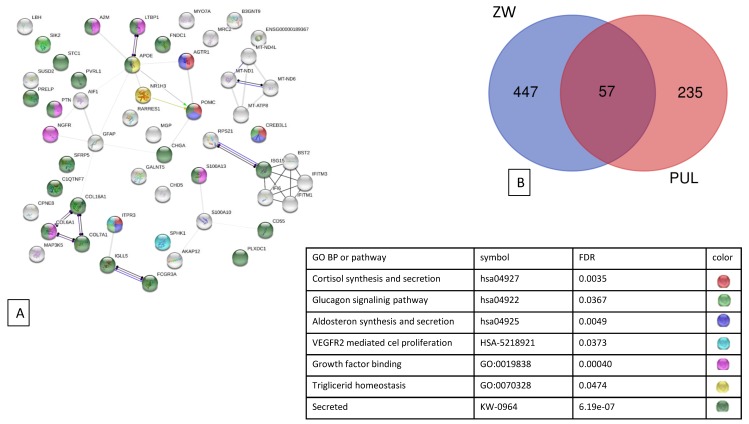
The string network of proteins encoded by identified DEGs in the (**A**) pituitary of Puławska (PUL) and Złotnicka White (ZW) pigs in response to variable feed conversion. (**B**) A Venn diagram presenting DEGs identified for Puławska and Złotnicka White pigs with 57 shared genes.

The comparison of the DEGs between Puławska and Złotnicka White breeds showed 57 shared genes that encode proteins involved in growth factor binding, glucagon signalling, cortisol synthesis, secretion, and triglyceride homeostasis (Figure 2).

The pituitary responses related to changes in feed conversion indicated approximately 300 DEGs in Puławska pigs that encode proteins involved in the corticotropin-releasing factor receptor signaling pathway (*POMC, GNA14*), the negative regulation of the canonical Wnt signaling pathway (*TLE2, FRZB, TLE1, SFRP5*), the positive regulation of cholesterol transport (*LXRA, APOA1, APOE, ANXA2*), the regulation of hormone levels (*CYP1B1, APOA1, ANO1*, *AQP1, NR1D1, CRYM, DDO, SPP1, ARRB1, SOX8, BIRC5, CLCN2*), a response to oxidative stress (*CAT, MAP3K5, CYP1B1, APOD, SPHK1, APOE, NR4A2, AIF1, MT3, NOR-1, RBPMS*), and regulation of the insulin-like growth factor (IGF) transport and uptake by insulin-like growth factor binding proteins (IGFBPs) (*CHRDL1, SPP1, C4A, LTBP1, APOE, IGFBP6, IGF2*) (Table S2, https://bit.ly/2Wtbqlb). In turn, Złotnicka White DEGs were involved in the integrin signalling pathway (*LAMB2, ITGAE, MAP3K5, COL16A1, TLN1, VCL, COL4A2, COL5A1, COL12A1, FLNA, COL1A2, ITGBL1, LAMC1, COL1A1, LAMB1, ITGA6, BCAR1, COL6A3, ITGA3, ARPC3, ACTN4, NRAS, LAMA5*); positive regulation of cholesterol efflux (*LRP1, APOE, PTCH1, NR1H3, ABCG1, ABCA1*); gene silencing (*DNMT3A, AGO2, UBE2B, DHX9, TNRC6A, RIF1, ARID1A, TNRC6C, DNMT1, GIGYF2, DICER1*); positive regulation of the Notch signaling pathway (*NOV, MAML3, AAK1, NOTCH1, ROBO1, ERH, ZMIZ1, KIT, EP300, CREBBP, PDCD10*), the Wnt signaling pathway (33 genes), and the response to hormones (71 genes) (Table S3, https://bit.ly/2Wtbqlb).

**Figure 3:**
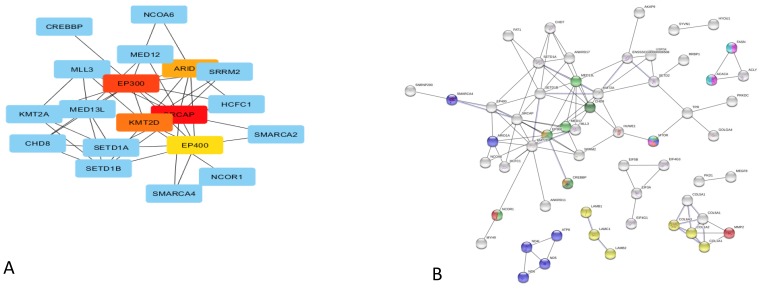

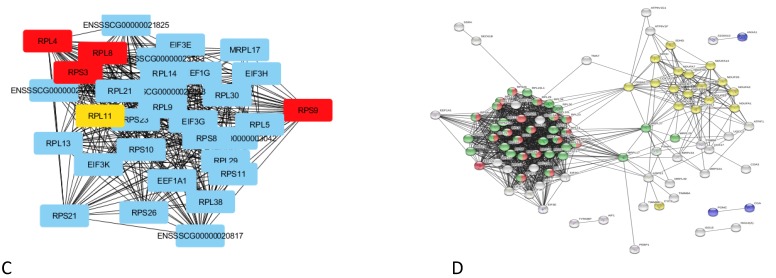
Co-expression analysis of the DEGs in Złotnicka White pigs. The gene network after Cytoscape, featuring a CytoHubba plugin analysis with the top five nodes ranked by the MCC test for high (**A**) and low (**C**) feed conversion pigs. The gene co-expression network predicted by STRING with the indicated metabolic pathway involvement in high (**B**) and low (**D**) feed conversion pigs. Legend: 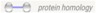, 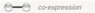, high feed conversion Złotnicka White pigs: 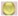 ECM receptor interaction (ssc04512), 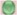 the thyroid hormone signaling pathway (ssc04919), 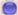 thermogenesis (ssc04714), 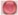 endocrine resistance (ssc01522), 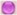 the AMPK signaling pathway (ssc04152), 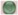 the Wnt signaling pathway (ssc04310), 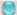 the Insulin signaling pathway (ssc04910), 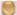 the Notch signaling pathway (ssc04330); low feed conversion Złotnicka White pigs: 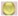 non-alcoholic fatty liver disease (NAFLD) (ssc04932), 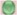 ribosome (ssc03010), 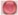 GO:0006412 translation, and 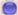 GO:0010817 regulation of hormone levels.

**Figure 4:**
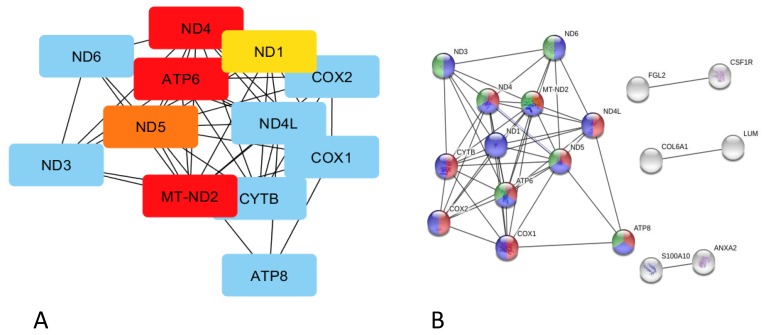
Co-expression analysis of genes overexpressed in Puławska pigs, characterized by high feed conversion values. (**A**) The gene network after Cytoscape using a CytoHubba plugin analysis with the top five nodes ranked by the Maximal Clique Centrality (MCC) test. (**B**) The gene co-expression network predicted by STRING, indicating the pathway involvement. Legend: 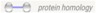, 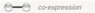, 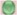 respiratory electron transport, ATP synthesis by chemiosmotic coupling, and heat production by the uncoupling proteins (Reactome pathway SSC-163200), 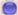 thermogenesis (ssc04714), 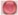 and the ATP metabolic process (GO:0046034).

The Cytoscape (Cytohubba plugin) and STRING indicated genes that were co-expressed in each of the analysed pig groups (Figure 3 and Figure 4). The analysis identified *EP300* as a hub gene correlated with elevated fat deposition and high feed conversion values in the pituitary of Złotnicka White pigs. The *EP300* was co-expressed with the other genes involved in Notch1 (*CREBP*), Wnt (*CREBP*, *MTOR*, and *CHD8*), and thyroid hormones (*NCOR1, MED12, MED13L*, and *CREBP*) signalling pathways. Moreover, the co-expression analysis showed that several co-transcripted genes play a role in thermogenesis; these genes include *ATP8, ND5, ND6, ND4L, ARDI1A*, and *SMARCA4*. In low FC Złotnicka White pigs, co-expressed genes were associated with ribosome biogenesis and the translation process. The STRING network also reveals that *POMC* and *CGA* co-expression is involved in hormonal regulation. In Puławska pigs, co-expressed genes positively correlated with feed conversion to play a role in thermogenesis, respiratory electron transport, and ATP metabolism processes. The co-expression analysis between the DEGs in LFC Puławska pigs did not find any connections.

### 3.3. qPCR Analysis

**Figure 5:**
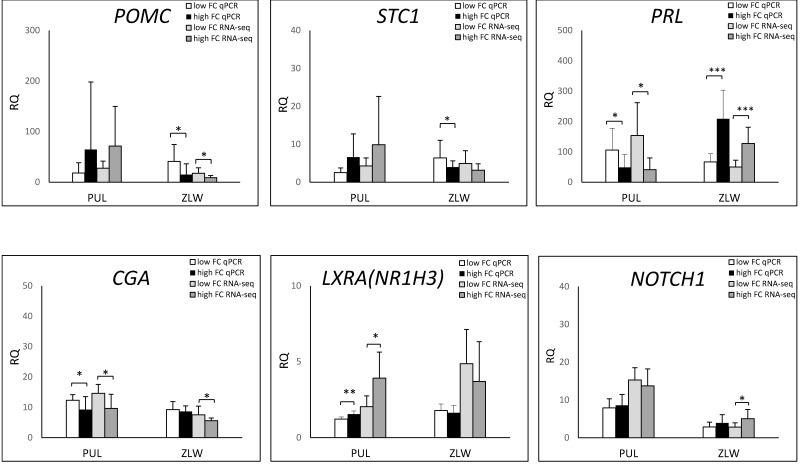
The relative quantification of the DEGs involved in the regulation of hormonal levels, as identified by RNA-seq. The relative transcript abundance of genes evaluated in the pituitary of low (*n* = 8) and high feed conversion (FC) (*n* = 8) Puławska and Złotnicka pigs. The *RPS29* and *OAZ1* genes have been used as endogenous controls. The efficiency of the PCR reactions was estimated based on the standard curve method. The gene expression levels were calculated using the delta–delta CT method [19]. Significant differences between gene expression levels were determined by ANOVA. *POMC*—pro-opiomelanocortin, *STC1*—stanniocalcin-1, *PRL*—prolactin, *CGA*—glycoprotein hormones, *LXRA (NR1H3)*—nuclear receptor subfamily 1 group H member 3, and *NOTCH1*—notch receptor 1.

The relative quantification for seven DEGs (*POMC, NOTCH1, CGA, NR1H3, PRL, RYR2*, and *STC1*) was estimated by the qPCR method. The lowest R coefficient (0.39) obtained for the *NR1H3* gene. This observation was likely the consequence of numerous isoforms encoded by this gene. The R coefficients for the other genes were 0.97, 0.81, 0.84, 0.91, 0.89, and 0.80 for *POMC, NOTCH1, CGA, PRL, RYR2*, and *STC1,* respectively. The qPCR analysis confirmed the RNA-seq results. The comparison of the RQ levels between Puławska and Złotnicka White pigs showed an opposite tendency in *POMC* expression, depending on the pig breed. For Puławska pigs, *POMC* expression correlated with increased FC. For Złotnicka White pigs, an elevated *POMC* mRNA level was observed in the faster-growing animals. The same tendency was recorded for *PRL, STC1*, and *CGA* genes (Figure 5).

## 4. Discussion

Feed conversion has become one of the most critical factors in animal production, including pig farms. The present study, using next-generation sequencing, shows the candidate genes and biological processes that are induced in the pituitary response to variable feed conversion. The pituitary is one of the most essential glands, as it regulates the body’s water level, temperature, heartbeat, urination, sleep, hunger, and thirst [4]. Therefore, most processes activated in this organ translate into phenotypic traits and bodily functions.

### 4.1. Inducing of Pituitary Gene Expression in Response to Variable Feed Conversion in Pigs

The two pig breeds involved in the present investigation, although they belong to native populations without selection pressure, often displayed opposite pituitary responses dependent on feed conversion. This state could be related to differences in their growth process and the increased fat content in Złotnicka White pigs. Nevertheless, in both breeds, numerous shared DEGs were identified to be involved in cholesterol efflux, lipid metabolism (regulated by peroxisome proliferator-activated receptor-alpha), and cholesterol transport. Among these DEGs were two relevant to lipid metabolism, *LXRA* and *APOE.* The liver X receptor alpha (LXRA) regulates cholesterol, lipid, and glucose metabolism [20], and its impaired protein function implicates it in diabetes and cardiovascular disease. Although the *LXRA* mRNA level was low in the pituitary, the present study found the differences in its gene expression to be dependent on feed conversion values. Within Puławska pigs, increased *LXRA* expression favoured higher FC values, with the opposite tendency in Złotnicka White pigs. Nilsson et al. [20] identified that the LXR agonist treatment-induced POMC secretion in the pituitary cell culture. The authors suggested that LXRs independently interfere with hypothalamic–pituitary–adrenal axis regulation. In turn, Christoffolete et al. [21] suggested that LXRA controls feed intake, which is associated with lipid metabolism. Yu et al. [22] showed that *LXRA* polymorphisms affected the fat levels in pig carcasses. In turn, Piórkowska et al. [23], who identified the candidate genes for meat quality in pigs, found that increased *LXRA* expression was negatively associated with feed efficiency and daily gains. 

The apolipoprotein E (APOE) function was examined using Apoe^−/−^ mice [24]. The lack of *APOE* disturbed the hypothalamic–pituitary–adrenal axis mediated by the glucocorticoid, which plays a role in various brain functions, such as cognition, emotion, and feeding. In another study, Babenko et al. [25] supported the thesis that *APOE* can be the driver of the neighbouring genes’ expression alteration observed under stressful loads. Moreover, the authors pinpointed that APOE and POMC in tandem is essential in specific energy metabolism and signalling cascades in the brain. The present study confirmed this observation because pro-opiomelanocortin (*POMC*) expression in the pituitary was positively correlated with *APOE* and *LXRA* gene expression levels, as well as with feeding stimuli in Puławska pigs. 

The hypothalamic POMC is cleaved via prohormone convertase (PCs) into smaller peptides [26] with anorexigenic activities (α-MSH and β-MSH) [27,28] and orexigenic abilities, such as β-endorphin [29]. In the pituitary, pro-opiomelanocortin decays to the adrenocorticotropic hormone (ACTH), which affects gluconeogenesis by releasing, from the adrenal cortex, glucocorticoids (GCs) that enhance the availability of glucose. This energy mobilization promotes the loss of body mass, which has also been observed in the investigated Puławska pig population. In pigs with increased feed conversion and feed intake, as well as lowered daily gains, elevated *POMC* expression followed. These findings are consistent with the experiment of the GC administration, which promoted ad libitum food intake both in humans and animals [30]. The role of *POMC* in Złotnicka White pigs seems to be different from that in the Puławska pig population, since its expression was negatively related to FC and body mass loss. In Złotnicka White pigs, *POMC* expression did not also correlate with feed intake. On the other hand, the Cytoscape and STRING analyses identified that *POMC* co-expressed with the *CGA* gene in the pig pituitaries. The *CGA* encodes the glycoprotein hormone; it is also crucial in regular TSH activity and likely increases the circulating levels of the thyroxine (T4) hormone [31]. Martin et al. [32] showed a reduction in the production of pituitary TSH and hypothalamic TRH in *POMC* KO (knockout) mice. This observation could be associated with the mediating role of CGA in TSH functionality.

The present study also showed increased prolactin (*PRL*) expression to be positively correlated with fat content in Złotnicka White pigs (confirmed by qPCR analysis). Numerous studies have described prolactin’s functions in terms of their effects on lactation and reproduction [33,34]. Prolactin secretion is regulated by dopamine, which was demonstrated using mice lacking the dopamine D2 receptor and exhibiting hyperprolactinemia [35]. Dopamine is believed to be an anorexigenic molecule because when released in the ventral tegmental area of brain, it suppresses feed intake [36]. The present study identified increased *PRL* expression in more obese Złotnicka White pigs. This finding is consistent with a previous survey of patients with hyperprolactinemia with increased fat content and reduced lean mass. The present Cytoscape analysis did not detect any co-expression for the *PRL* gene.

### 4.2. The Enriched Insulin-Like Growth Factor Pathway

The previously mentioned *APOE* also plays a role in the regulation of the insulin-like growth factor (IGF) transport and uptake by the insulin-like growth factor binding protein (IGFBP) pathway. The present study identified several other DEGs (*IGF2, IGFBP6, IGFBP5*, and *IGF1R*) to be associated with this pathway. These genes encode proteins belonging to the IGF family, which consists of ligands (insulin, IGFs), binding proteins (IGFBPs), and cell surface receptors (IGF1R, IGF2R, and IR) [37]. *IGF2* is an imprinted gene with an expressed paternal allele. It controls foetal growth [38], but significant evidence shows its essential function during postnatal development. IGF-II is critical for amplifying and maintaining myoblast determination protein 1 (MyoD) efficacy during muscular development [39]. It also promotes the proliferation and differentiation of bone cells [40]. The present study also showed increased *FOXO3* expression in the pituitary glands of obese Złotnicka White pigs. The *FOXO3* gene was proposed as a candidate for chicken growth [41] because silencing this gene in a chicken fibroblast culture significantly inhibited MyoD and up-regulated growth-related genes, such as *INSR, GH*, and *IGF2Rs*. Therefore, the *FOXO3* gene is considered to be related to the IGF family. Returning to *IGF2*, in pigs, it was found that intron mutation *IGF2* G>A3072 disrupts a binding site for the repressor zinc finger BED-type containing 6 (ZBED6), which results in an elevation of *IGF2* expression that is associated with increased meat content in the carcass [42,43]. In the present study, augmented *IGF2* expression in the pituitary of Puławska pigs with an increased daily feed intake and low daily gain values was observed. Thus, pituitary *IGF2* expression did not positively relate to growth in 6 month old pigs. The present Cytoscape analysis did not detect any co-expression for the *IGF2* gene.

### 4.3. The Enriched Notch Signalling Pathway

**Figure 6:**
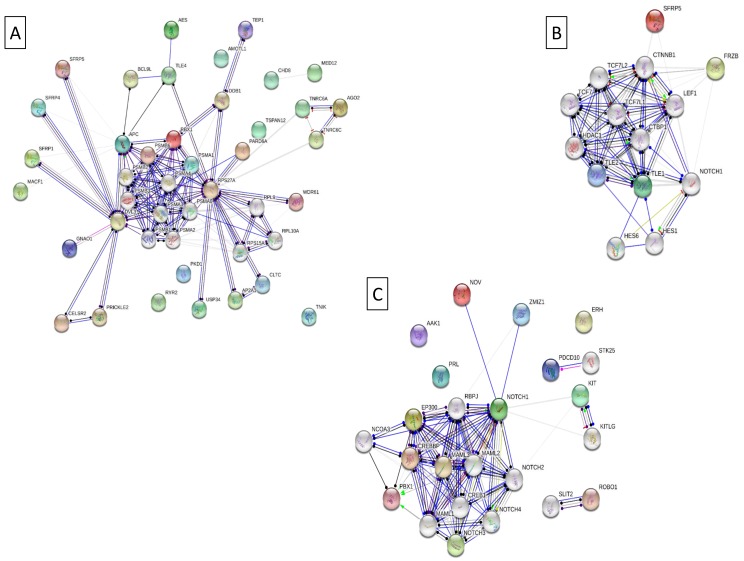
The string network of proteins (encoded by the differentially expressed genes (DEGs)) involved in signalling pathways. They enriched (**A**) the Wnt signalling pathway in Złotnicka White pigs, (**B**) the negative regulation of the canonical Wnt signalling pathway in Puławska pigs, and (**C**) the positive regulation of the Notch signalling pathway in Złotnicka White pigs. The coloured orbs indicate DEGs, and the grey orbs are the backgrounds.

Notch signalling was induced in response to variable feed conversion in Złotnicka White gilts that were more obese and were characterized by poor feed efficiency (Figure 6). The Notch signalling pathway is essential for pituitary proliferation and progenitor maintenance in postnatal pituitary expansion [44]. A study using Notch2 cKO mice showed that pituitary *GH, TSHB, PRL* and *POU1F11(Pit1*) expressions are strongly dependent on Notch signalling through the transcription factor, PROP1. Moreover, in Notch2 cKO mice, increased *POMC* expression was observed to be consistent with the present results, where Notch signalling negatively correlates with *POMC* expression in Złotnicka White pigs. Numerous studies have proven that Notch signalling promotes obesity because it boosts gluconeo- and lipogenesis in the liver, leading to fatty liver disease [45]; induces the whitening of brown adipose tissue and insulin resistance in adipocytes [46]; and increases fat deposition in the hypothalamus by the activation of B cells (NF-κB) in mice on a high-fat diet [47]. The present study supports the positive effect of Notch signalling on fat content since Notch signalling was activated only in Złotnicka White pigs characterized by a high body fat level. Additionally, the CytoHubba analysis found that the *CREBBP* and *EP300* genes involved in Notch signalling are co-expressed. *CREBBP* and *EP300* are highly homologous genes that play essential roles as global transcriptional coactivators [48]. In the present study, *CREBBP* and *EP300* overexpression was correlated with slower growth and increased fat content in pigs. CREBBP is the binding protein for the cAMP-responsive element-binding protein (CREB), which is the critical regulatory element for hypothalamic–pituitary axis development because crucial transcription factors in the pituitary have a CREB binding site in their promoters. Firstly, CREB [49] plays a role in the production of pituitary GH, which was demonstrated using genetically engineered mice, where decreased CREB activity resulted in dwarfism [50], and increased cAMP levels led to pituitary hyperplasia and gigantism [51]. The present study also found two CREB-like protein genes in the DEG group (*CREB3L2* and *CREB3L1*), but to date, their role remains unclear. In turn, the mutations in *CREBBP* and *EP300* have been described in Rubinstein–Taybi syndrome, where children have growth disturbances in the first few months, resulting in short statures [48]. Thus, *CREBBP* and *EP300* could be crucial in pig growth and feed conversion.

### 4.4. The Enriched Wnt Signalling Pathway

Wnt signalling likely plays a significant role during growth, fat accumulation, and feed intake in pigs. The present study found enriched Wnt signalling in ZW pigs with increased fat mass and weak feed efficiency. However, in Puławska pigs, the genes associated with negative regulation of the canonical Wnt signalling pathway were overexpressed (Figure 6). In turn, the CytoHubba analysis showed that *EP300, CREBBP, MTOR*, and *CHD8* involved in Wnt signalling were co-transcribed. EP300 and CREBBP were described, previously, as critical transcriptional regulators. In turn, the *mTOR* (mammalian target of rapamycin) pathway plays a central role in the regulation of cell growth, metabolism, and apoptosis. In the pituitary, *MTOR* is involved in the PI3K/AKT/mTOR signalling pathway traditionally described as a pro-proliferative and pro-survival pathway [52]. 

Wnt signalling is also involved during embryogenic development [53] and is essential in brain morphogenesis and function [54]. In turn, recent studies indicate that Wnt signalling is critical for neuroendocrine control in the hypothalamus. Wnt signalling is also linked with a risk of type 2 diabetes because it can bind to the co-activator of β-catenin TCF7/L2 [55], and the Wnt/β-catenin pathway is responsive to glucose [56]. The beta-catenin in the hypothalamus also affects NPY and POMC neurons, which suggests that the Wnt pathway regulates obesity by controlling food intake behaviour [57]. The present study showed that the secreted frizzled-related proteins (SFRPs) associated with Wnt signalling are differentially expressed in pig pituitaries, dependent on variable feed conversion. Increased *SFRP1* and *SFRP4* expressions in obese and lean Złotnicka White pigs have also been observed, respectively. In turn, *SFRP5* expression is positively correlated with lean meat in Złotnicka pigs and slower growth in Puławska pigs. The role of SFRP proteins in the pituitary is not precisely clear, but their decreased expression was observed during tumorigenesis [58]. In the pituitary, the Wnt signalling pathway is not involved in the regulation of feed intake like in hypothalamus, but it plays an essential role during gland development and maturation and stimulates cell proliferation during tumorigenesis [58].

## 5. Conclusions

In summary, feed conversion is a highly economically important trait in farm animals that determines animal maintenance costs. However, feed conversion is an exponent of many factors. Thus, for Złotnicka White pigs, lowered feed conversion values were associated with increased fat content, while in the Puławska pig population, these values were associated with slower growth. Therefore, the pituitary response to variable feed conversion was extremely different between these breeds. Nevertheless, the current research shows a significant relationship between hormonal pituitary activity and feed conversion determination. Here, we presented several candidate genes, such as *POMC, PRL, EP300, CREBBP, APOE, IGF2*, and *CGA*, that are differentially expressed in the pituitary in response to variable feed conversion in pigs. These genes play significant roles in the crucial pituitary processes that determine pig phenotypes.

## Figures and Tables

**Table 1:** The differences in porcine traits observed between the analysed groups for both breeds.

Trait	Group of Native Pigs			
	Puławska		Złotnicka White	
	LFC (High Benefit) *n* = 8	HFC (Low Benefit) *n* = 8	LFC (High Benefit) *n* = 8	HFC (Low Benefit) *n* = 8
	Mean ± SD	Mean ± SD	Mean ± SD	Mean ± SD
Feed conversion ratio	2.57 ± 0.06 ^A^	3.64 ± 0.37 ^B^	2.84 ± 0.71 ^A^	4.12 ± 0.89 ^B^
Daily gain (g)	919 ± 103 ^A^	715 ± 82 ^B^	811 ± 98 ^A^	561 ± 165 ^B^
Daily feed intake (kg/day)	2.35 ± 0.27 ^a^	2.60 ± 0.23 ^b^	2.30 ± 0.12	2.32 ± 0.16
Day in test	78 ± 9 ^A^	95 ± 10 ^B^	84 ± 15 ^A^	122 ± 28 ^B^
Back ham (kg)	11.1 ± 0.71 ^a^	10.3 ± 0.61 ^b^	10.2 ± 0.51	9.8 ± 0.58
Ham (kg)	9.3 ± 0.64	8.74 ± 0.60	8.21 ± 0.59 ^A^	7.34 ± 0.65 ^B^
Ham fat (cm)	1.57 ± 0.13 ^a^	1.38 ± 0.21 ^b^	1.76 ± 0.21 ^a^	2.22 ± 0.38 ^b^
Backfat (cm) *	1.28 ± 0.44 ^a^	1.64 ± 0.19 ^b^	1.7 ± 0.47	1.64 ± 0.53
Knuckle fat	0.20 ± 0.01	0.20 ± 0.01	0.20 ± 0.01 ^a^	0.23 ± 0.01 ^b^
Backfat in C1 point *	1.30 ± 0.38	1.03 ± 0.28	1.53 ± 0.40 ^a^	2.03 ± 0.41 ^b^
Backfat in the K1 point *	1.30 ± 0.38	1.06 ± 0.26	1.53 ± 0.39 ^a^	2.01 ± 0.40 ^b^
%meat in primary cuts	70 ± 2.2	70 ± 2.4	66 ± 3.2 ^A^	61 ± 3.7 ^B^
%meat in carcass	61.4 ± 1.82	60.7 ± 2.85	57.5 ± 3.0 ^A^	52.9 ± 3.7 ^B^

Backfat *—backfat thickness was measured on the back above the joint between last thoracic vertebrae and the first lumbar vertebra. * Backfat in C1 point was measured between the last thoracic vertebrae and the first lumbar vertebra, 8 cm from away from the midline. * Backfat in K1 point was measured between the last thoracic vertebrae and the first lumbar vertebra above the lateral edge of the M. longissimus dorsi. LFC—low feed conversion, HFC—high feed conversion. Values with the same superscripts belong to the same statistical group (A, B = *p* < 0.01, a, b = *p* < 0.05).

## Data Availability

Data available in the GEO database: GSE122343—(https://www.ncbi.nlm.nih.gov/geo/query/acc.cgi?acc=GSE122343), GSE132522—(https://www.ncbi.nlm.nih.gov/geo/query/acc.cgi?acc=GSE132522), and under the link—https://bit.ly/2Wtbqlb. For the others, please contact the authors.

## Supplementary Materials

The following are available online at https://www.mdpi.com/2073-4425/10/9/712/s1, Figure S1: Bar plots show overall statistics and read annotations obtained for each library. Table S1: Primers and probes used in qPCR analysis. Table S2: DE pituitary gene dependent on feed efficiency/conversion based on Fisher exact test in Panther classification system for Puławska pigs.
