# Supplementary material for: The Pituitary Transcriptional Response Related to Feed Conversion in Pigs
Source: Genes (Basel). 2019 Sep 14;10(9):712. doi: 10.3390/genes10090712 (PMC6771146; doi:10.3390/genes10090712)
Supplement: Supplementary file 1 [file genes-10-00712-s001.pdf]

Table S1. Primers and probes used in qPCR analysis

Gene		Primers and Probe or Taqman Gene Expression Assay ID	Amplicon length [bp]	Label	Reference sequences accesion numbers	PCR efficiency %	Exon Boundary
PRL	prolactin	Ss03390993_m1	79	FAM	NM_213926.1	100	3-4
RYR2	ryanodine receptor 2 (cardiac)	Ss03374536_m1	62	FAM	U15966.1	100	4-5
CGA	Thyroid-Stimulating Hormone Alpha Chain	Ss03394998_m1	75	FAM	NM_214446.1	100	2-3
POMC	Proopiomelanocortin	Ss03381950_u1	158	FAM	NM_213858.1	100	3-3
NOTCH1	Notch 1	Ss03377164_u1	71	FAM	EF055896.1	100	-
NR1H3 (LXRA)	nuclear receptor subfamily 1, group H, member 3	Ss03389237_g1	101	VIC	NM_001101814.1	100	5-6
GAPDH	glyceraldehyde-3-phosphate dehydrogenase	Ss03375629_u1	61	VIC	NM_001206359.1	100	-
RPS29	ribosomal protein S29 **endogenous**	Ss06942053_g1	71	VIC	NM_001001633.1	100	1-2
STC1	stanniocalcin 1	Ss03389414_m1	59	FAM	NM_001103212.1	100	3-4
OAZ1	ornithine decarboxylase antizyme 1 **endogenous**	Ss03387505_u1	77	VIC	NM_001122994.1	100	5-5

Statistics

REMAINING_READS

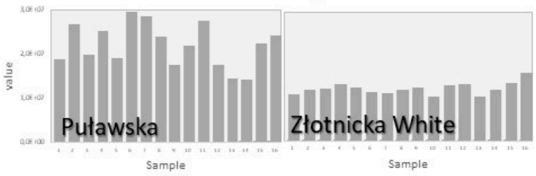

NUMBER_OF_UNIQUELY_MAPPED_READS

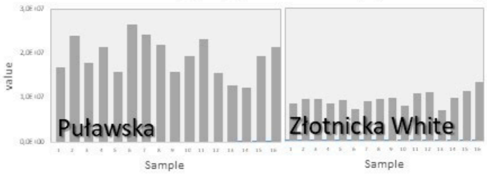

INTRAGENIC_RATE

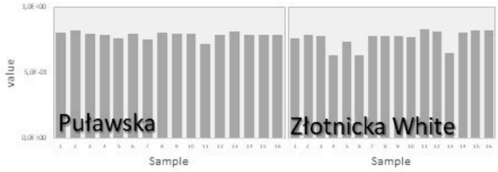

EXONIC_RATE

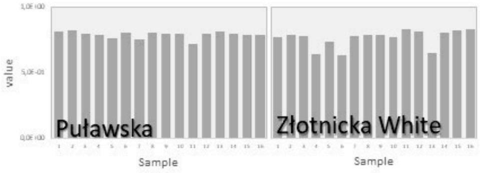

GENES_DETECTED

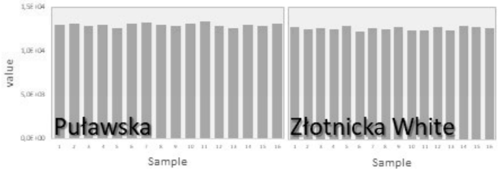

MAPPED

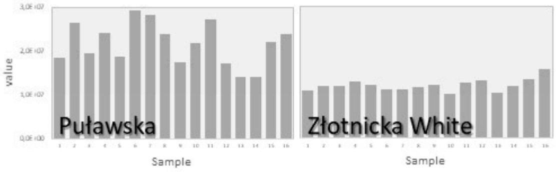

TRANSCRIPTS_DETECTED

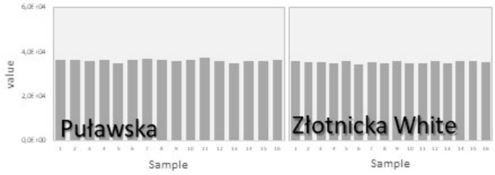

INTRONIC_RATE

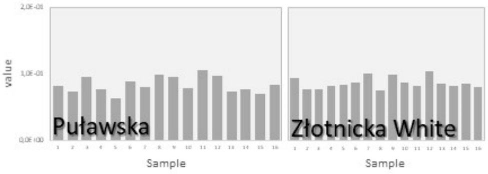

ALTERNATIVE_ALIGMENTS

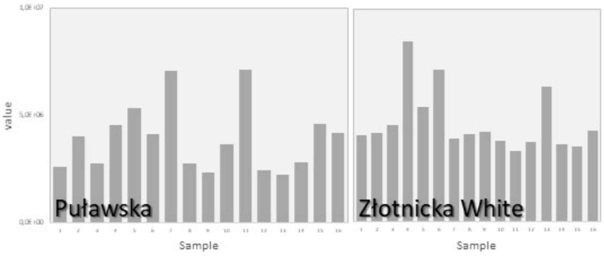

Table S2. DE pituitary gene dependent on feed efficiency/conversion based on Fisher exact test in Panther classification system for Puławska pigs

Gene ontology	FDR	No.	Genes
**PANTHER Pathways**			
Angiotensin II-stimulated signaling through G proteins and beta-arrestin	5.95E-03	5	*AGT, GNG11, AGRT1, ITPR3, ARRB1*
Alzheimer disease-presenilin pathway	6.96E-08	6	*CD44, MMP28, NECTIN1, FZD5, FSTL1, LRP4*
Cortocotropin releasing factor receptor signaling pathway	4.61E-02	2	*POMC, GNA14*
**GO-Slim Biological Process**			
negative regulation of canonical Wnt signaling pathway	4.33E-02	4	*TLE2, FRZB, TLE1, SFRP5*
cellular response to peptide hormone stimulus	4.61E-02	5	*NR4A1, AGTR1, NR4A2, PIK3R1, NOR-1*
**GO Biological Process Complete**			
mammary gland specification	4.54E-02	2	*FGF10, GLI3*
positive regulation of Rho protein signal transduction	2.77E-02	3	*APOA1, ADGRG1, ARRB1*
positive regulation of cholesterol transport	1.11E-02	4	*LXRA, APOA1, APOE, ANXA2*
retina development in camera-type eye	2.13E-02	7	*CYP1B1, NECTIN1, NTRK2, MERTK, SOX8, CLIC4, CLCN2*
regulation of G protein-coupled receptor signaling pathway	4.76E-02	6	*PLCE1, RAMP2, RAMP3, ARRB1, C3, ADM*
response to steroid hormone	6.95E-03	9	*LXRA, ACSBG1, SLIT3, NR1D1, NR4A1, ATP1A2, CAV1, NR4A2, NOR-1*
regulation of lipid biosynthetic process	3.23E-02	7	*HSD17B13, NR1D1, SIK1, FGF1, APOE, C3, CLCN2*
regulation of cellular response to growth factor stimulus	6.44E-03	11	*CREB3L1, AGT, FGF10, CHRDL1, SFRP5, FGF1, JCAD, CAV1, LTBP1, FOLR1, MT3*
sensory organ morphogenesis	1.16E-02	11	*GDF11, FGF10, NECTIN1, FRZB, AQP1, MYO7A, NTRK2, SOX8, NOR-1, FZD5, GLI3*
response to oxidative stress	1.19E-02	11	*CAT, MAP3K5, CYP1B1, APOD, SPHK1, APOE, NR4A2, AIF1, MT3, NOR-1, RBPMS*
kidney development	2.35E-02	10	*GDF11, PLCE1, AGT, FGF10, AQP1, FGF1, WNK4, SOX8, GLI3, LRP4*
regulation of MAP kinase activity	2.75E-02	10	*PLCE1, MAP3K5, FGF10, FGF1, MAP3K20, CAV1, APOE, FZD5, FGD2, DUSP6*
cellular response to lipid	1.91E-02	13	*LXRA, CX3CR1, AXL, NR1D1, NR4A1, ATP1A2, SPP1, IRF8, CCL2, NR4A2, RAMP3, NOR-1, VIM*
developmental growth	1.91E-02	12	*FGF10, SLIT3, APOD, S1PR1, FGF1, GJA1, GLI3, LRP4, SEMA7A, STC1, CLIC4, ADM*
secretion	4.64E-02	12	*FGF10, AXL, AQP1, CA2, S100A13, NTRK2, SYT11, GJA1, CAV1, MERTK, NOR-1, CHGA*
positive regulation of MAPK cascade	3.19E-02	13	*PLCE1, MAP3K5, FGF10, FGF1, JCAD, MAP3K20, APOE, RAMP3, ARRB1, MT3, FZD5, FGD2,*
regulation of hormone levels	4.88E-02	12	*CYP1B1, APOA1, ANO1, AQP1, NR1D1, CRYM, DDO, SPP1, ARRB1, SOX8, BIRC5, CLCN2*
apoptotic process	1.74E-02	14	*ZNF385A, MAP3K5, CYP1B1, 06-16, SLIT3, NR4A1, SIK1, CAV1, ARRB1, MT3, BIRC5, PIK3R1,*

regulation of secretion	3.61E-02	14	*LXRA, FGF10, APOA1, CSF1R, OAS2, ANG, ANO1, NR1D1, SPP1, SYT11, WNK4, ARRB1, AIF1,*
positive regulation of gene expression	8.07E-03	35	*CREB3L1, LBH, MAP3K5, AGT, FGF10, LUM, POMC, SPON1, TLE1, NR1D1, NR4A1, RAMP2, FAM46C, SPP1, FGF1, IRF8, NTRK2, GJA1, CAV1, FOSL2, APOE, NR4A2, RAMP3, ARRB1, MT3, SAMD4A, SOX8, ARHGEF10L, PIK3R1, NOR-1, FZD5, NFIX, GLI3, NGFR, VIM*
REACTOME Pathways			
Regulation of Insulin-like Growth Factor (IGF) transport and uptake by Insulin-like Growth Factor Binding Proteins (IGFBPs)	3.40E-02	7	*CHRDL1, SPP1, C4A, LTBP1, APOE, IGFBP6, IGF2*

Abbreviations: NA-nucleic acid, : FDR – false discovery rate, No. – number of identified genes involved in GO, GO-gene ontology

Table S3. DE pituitary gene dependent on feed efficiency/conversion based on Fisher exact test in Panther classification system for Złotnicka White pigs

Gene ontology	FDR	No.	Genes
PANTHER Pathways			
Integrin signalling pathway	9.44E-03	23	*LAMB2, ITGAE, MAP3K5, COL16A1, TLN1, VCL, COL4A2, COL5A1, COL12A1, FLNA, COL1A2, ITGBL1, LAMC1, COL1A1, LAMB1, ITGA6, BCAR1, COL6A3, ITGA3, ARPC3, ACTN4, NRAS, LAMA5*
PANTHER GO-Slim Molecular Function			
nuclear hormone receptor binding	2.53E-02	5	*NCOA3, NCOR2, JUP, NCOR1, NCOA2*
G-protein coupled receptor activity	9.86E-18	5	*PTGFR, ANXA1, AGTR1, GNAO1, CXCR4, CHGA*
PANTHER GO-Slim Biological Process			
oxidative phosphorylation	3.36E-03	16	*NDUFS6, ENSSSCG00000003903, COX7A1, CYCS, COX7A2, NDUFA7, UQCRB, MT-ND5, NDUFB7, SDHD, COX7C, NDUFB5. SDHC. COX6A1*
GO biological process complete			
positive regulation of cholesterol efflux	4.56E-02	5	*LRP1, APOE, PTCH1, NR1H3, ABCG1, ABCA1*
regulation of cardiac muscle cell contraction	1.56E-02	7	*ANK2, CACNA1C, SRI, JUP, AKAP9, STC1, RYR2*
gene silencing	3.63E-02	11	*DNMT3A, AGO2, UBE2B, DHX9, TNRC6A, RIF1, ARID1A, TNRC6C, DNMT1, GIGYF2, DICER1*
cellular response to oxidative stress	1.23E-02	17	*MGST1, MAP3K5, PRDX5, KDM6B, EPAS1, RPS3, ZNF277, MMP2, SPHK1, CHD6, GPX1, AIF1, SELENOS, ATP2A2, FOXO3, PDCD10, EGFR*
regulation of cellular response to growth factor stimulus	2.98E-02	21	*CREB3L1, FLT1, EP300, SFRP4, SFRP1, NUMA1, CREBBP, CAV2, MYOF, SFRP5, ENG, MYO1C, NOTCH1, LTBP1, SLIT2, ITGA3, GLG1, SULF1, BCL9L, PRKD2, ROBO1*
muscle structure development	3.23E-02	29	*MEF2D, DYSF, EP300, CHD2, MSC, MYH14, FLNC, KDM6B, EPAS1, CHD7, CAV2, MYOF, ARID1A, ENG, PLEKHO1, NOTCH1, LGALS1, MYH9, GPX1, IGFBP5, EGR1, HEG1, ATP2A2, MTOR, BCL9L, NF1, SLC9A1, LAMA5, RYR2*

heart development	8.85E-03	35	*SETD2, ANK2, PTCH1, MEF2D, CACNA1C, EP300, PKD1, CC2D2A, KDM6B, COL5A1, CHD7, PRKDC, ARID1A, FAT4, ENG, INSR, IFT172, MED12, NOTCH1, LTBP1, ID1, NOTCH2, MEGF8, GAA, ECE1, TENM4, HEG1, BCOR, SH3PXD2B, MTOR, ZMIZ1, NF1, SLC9A1, RYR2, ROBO1*
skeletal system development	3.09E-02	32	*SETD2, KIAA1217, CYP26B1, MEF2D, SLC38A10, EP300, DEAF1, PKD1, SFRP4, SFRP1, ASH1L, CHD7, NOV, MGP, COL1A2, ANKRD11, MMP2, FAT4, IFT172, RAI1, COL1A1, MED12, KIT, MEGF8, PBX1, GLG1, ZBTB16, ECM1, CREB3L2, SULF1, SH3PXD2B, STC1*
regulation of growth	3.29E-02	36	*PTCH1, SMARCA4, MAP1B, ZNF639, CDK4, CLSTN3, CGA, SFRP1, CHD7, PLXNA4, SAFB, NOV, RFTN1, TAF9, SPHK1, INSR, RAI1, NOTCH1, PTPRS, SELENOP, NOTCH2, SMARCA2, SLIT2, MEGF8, IGFBP5, PLAC8, PLXNA3, GOLGA4, PRL, SH3PXD2B, AGRN, WFS1, MTOR, EIF4G1, EGFR, MACF1*
response to insulin	2.59E-02	24	*PHIP, SELENOS, ATP6V1F, EIF3A, CAV2, CDK4, SOGA1, IGF1R, BCAR1, PRKDC, SREBF1, ACACA, CAD, INSIG2, TEP1, MTOR, FOXO3, ATP6V1G1, SLC9A1, EGR1, ATP6V0A1, INSR, GGH, APC*
positive regulation of transcription of Notch receptor target	2.16E-02	6	*NOTCH3, MAML3, NOTCH1, PRL, PBX1, EP300, CREBBP*
positive regulation of Notch signaling pathway	3.76E-03	11	*NOV, MAML3, AAK1, NOTCH1, ROBO1, ERH, ZMIZ1, KIT, EP300, CREBBP, PDCD10*
osteoblast differentiation	1.03E-03	19	*CLTC, MEF2D, FASN, MYBBP1A, RRBP1, HSPE1, GPNMB, SH3PXD2B, SNRNP200, NF1, IGFBP5, DHX9, MRC2, COL6A1, SPP1, SFRP1, COL1A1, RPS15, CREB3L1*
response to transforming growth factor beta	4.82E-02	18	*CBL, STK16, MXRA5, LTBP4, CDH5, PARD6A, COL4A2, RPS27A, ID1, PDGFD, ENG, LTBP2, DUSP15, ZFHX3, SFRP1, COL1A1, USP9X, COL1A2*
cellular response to hypoxia	2.55E-02	21	*DNMT3A, RBX1, HYOU1, NOTCH1, TEP1, MTOR, FAM162A, FAM162A, RPS27A, STC1, SLC9A1, EGR1, EP300, CREBBP, PSMA1, S100B, EPAS1, PSMB6, SFRP1, PDK3, PTN*
response to hormone	4.91E-03	71	*PTGFR, PHIP, SELENOS, ATP6V1F, DNMT3A, CBL, EIF2B1, PLOD3, ANXA1, EIF3A, CAV2, CDK4, SFRP4, NCOA3, EIF4E, ITGA3, SOGA1, IGF1R, BCAR1, AGRN, PRKDC, ATP1A1, SSR4, SREBF1, ACACA, CAD, NOTCH1, NCOA6, INSIG2, CAD, SF1, TEP1, ABCA2, MTOR, CGA, FOXO3, PTCH1, ATP6V1G1, NCOR2, STXBP1, STC1, IGFBP5, KIT, SLC9A1, NR1H3, EGR1, ARID1A, SAFB, AGTR1, PRL, ATP6V0A1, ENG, RGS10, CALM3, SPP1, S100B, INSR, GGH, ABCG1, SFRP1, COL1A1, AIF1, IQGAP1, PEBP1, NCOA2, HTT, APC, MDK, SLIT2, EGFR, PTN*
Wnt signaling pathway	2.64E-02	33	*RYR2, CLTC, AES, RBX1, SFRP4, USP34, CELSR2, DVL3, BCL9L, PARD6A, TEP1, PKD1, TLE4, MED12, AP2A1, CHD8, TSPAN12, RPS27A, MACF1, GNAO1, SFRP5, WDR61, TNRC6C, PRICKLE2, TNRC6A, PSMA1, DDB1, PSMB6, SFRP1, TNIK, APC, AGO2, AMOTL1*
Regulation of lipid metabolism by Peroxisome proliferator-activated receptor alpha (PPARalpha)	2.35E-02	15	*MED14 , NCOA3 , MED21 , MED13L , CHD9 , NCOA6 , CCNC , MED12 , NCOR2 , NR1H3 , NCOR1 , EP300 , CREBBP , NCOA2 , ABCA1*

Abbreviations: NA-nucleic acid, : FDR – false discovery rate, No. – number of identified genes involved in GO, GO-gene ontology
